# Experimental Murine Periodontitis Increases Salivary Gland IgA‐Producing B Cells Following Oral Dysbiosis

**DOI:** 10.1111/1348-0421.13191

**Published:** 2024-12-22

**Authors:** Mai Nara, Mie Kurosawa, Momoe Itsumi, Hirobumi Morisaki, Haruka Fukamachi, Nobuo Okahashi, Noriyuki Suzuki, Hirotaka Kuwata

**Affiliations:** ^1^ Department of Conservative Dentistry, Division of Endodontology Showa University Graduate School of Dentistry Ota‐ku Tokyo Japan; ^2^ Department of Oral Microbiology and Immunology Showa University Graduate School of Dentistry Shinagawa‐ku Tokyo Japan

**Keywords:** dysbiosis, IgA, mucosal immunology, oral microbiome, periodontal inflammation, salivary gland

## Abstract

The oral microbiome is closely involved in the maintenance of host health and the development of systemic diseases. The salivary glands play an essential role in homeostasis in the oral cavity. Here, we investigated the effects of periodontal inflammation on salivary gland function and the oral microbiome. In experimental periodontitis model mice, an increase in IgA⁺ cells in the salivary glands were observed 1 week after treatment. Alteration of the oral microbiome was also induced in this model. Gene expression analysis of the salivary glands showed changes in the expression of genes related to B‐cell maturation and plasma cell differentiation and an increase in the expression of genes related to macrophage activation upon experimental periodontitis induction. Furthermore, the relationship between disruption of oral microflora and salivary gland function was examined using a cohousing model in which experimental periodontitis model mice and untreated mice were reared in the same cage. We found that cohoused normal mice underwent alteration of the oral microbiome, with increases in IgA⁺ cells and macrophages in the salivary glands. In summary, our results suggest that, in the oral cavity, there is a close link between oral bacterial flora and immune cells in the salivary glands. Our results also show that localized inflammation disrupts the homeostasis in the oral cavity, inducing pathological conditions such as dysbiosis. Our study suggests the importance of the interaction among local oral inflammation, salivary gland function, and oral microflora, and provides new insights into the mechanisms by which oral health is maintained.

**Abbreviations**
AB: alveolar bone
BH: Benjamini–Hochberg
Calhm6: calcium homeostasis modulator family member 6
cLN: cervical lymph nodes
Ctrl: control group
D: dentin
Dcpp2: demilune cell and parotid protein 2
DEGs: differentially expressed genes
DMEM: Dulbecco's modified Eagle's medium
FBS: fetal bovine serum
G: gingiva
GO: gene ontology
HE: hematoxylin and eosin
Isoc2b: isochorismatase domain containing 2b
L1w: ligation for 1 week group
L2w: ligation for 2 weeks group
LEfSe: linear discriminant analysis effect size
LLP: large lamina propria
Ly6c2: lymphocyte antigen 6 family member C2
micro‐CT: micro‐computed tomography
NCo: non‐cohoused control group
PBS: phosphate‐buffered saline
PCA: principal component analysis
PFA: phosphate‐buffered formaldehyde
PM: periodontal membrane
Pou2af1: POU class 2 homeobox associating factor 1
Rcp: recipient mouse group
RLE: relative log normalization
SG: salivary glands
sIgA: secretory immunoglobulin A
Slfn4: schlafen 4
SS: Sjögren's syndrome
TBS: Tris‐buffered saline
Tnf: tumor necrosis factor
Tnfrsf13b: tumor necrosis factor receptor superfamily, member 13b
TPM: transcripts per million
Twist1: twist family bHLH transcription factor 1
Vpreb1: V‐set pre‐B cell surrogate light chain 1

## 1 Introduction

The bacterial flora of various human tissues, including the oral cavity, affects the immune function of the host, which is closely related to the maintenance of host health and the development of diseases [1, 2]. In recent years, attention has focused on how destabilization of the commensal microbiota can cause disease development. Abnormal microbiome composition, referred to as dysbiosis, is now considered to trigger various diseases. For example, studies on the interaction between the intestinal microbiota and the mucosal immune system have shown that symbiotic microorganisms such as *Clostridium* spp. in the intestinal microbiota produce short‐chain fatty acids such as butyric acid and maintain immune homeostasis by activating regulatory T cells and modulating B‐cell differentiation, thereby contributing to the maintenance of host health [3, 4].

In the oral cavity, it has also been shown that periodontal disease and other oral diseases are caused by oral dysbiosis, while systemic diseases such as diabetes, cardiac disease, and metabolic disorders are also exacerbated [5, 6]. The interaction between the oral microflora and the oral mucosal environment is thus important for the maintenance of oral health; the salivary glands in particular are considered to be important for maintaining oral homeostasis [7]. Salivary components include secretory immunoglobulin A (sIgA) as well as antibacterial substances such as lysozyme and lactoferrin. sIgA is mainly secreted by mucosal tissues such as the salivary glands and the intestinal tract and plays an essential role in the homeostasis of mucosal tissues such as the oral cavity and intestinal tract. Recent studies focusing on the intestinal tract suggest that sIgA plays a key role in maintaining a stable community of beneficial gut bacteria. It achieves this by binding to harmful microorganisms, inhibiting their growth, and eliminating foreign competitors, thereby promoting a balanced and stable bacterial colony in the gut [8, 9].

Previous studies have shown that the salivary glands respond acutely to changes in physical or mental status, altering the production of various bioactive substances [10, 11]. In fact, free cortisol and amylase in saliva are markers of stress responses. In addition, inflammation‐related changes in saliva, such as changes in the levels of cytokines, chemokines, or inflammation‐related proteins, have been observed upon the induction of periodontitis in both animal models and humans, suggesting that salivary glands also sense local inflammation in the oral cavity. However, the specific mechanism of these interactions has not been comprehensively elucidated [12, 13, 14, 15].

In a previous study [16], we quantitatively evaluated the compositional changes of the oral microflora by flow cytometry using a combination of antibodies against the cariogenic bacterium *Streptococcus mutans* surface antigen wapA as well as sIgA antibodies during the deterioration of oral hygiene. We found that the amount of binding of sIgA antibodies to bacteria changed with disruptive changes in the bacterial flora [16]. This suggests that changes in the oral microflora environment are monitored by the salivary glands, which maintain the homeostasis of the oral microflora by altering the substances that they secrete into the saliva.

In recent years, studies have been actively conducted to elucidate the interactions between the oral cavity and remote organs such as the intestinal tract [17, 18, 19]. These studies have shown that the transfer of oral bacteria to the intestinal tract and other organs results in endogenous infection and that oral bacteria are recognized as ectopic common antigens, causing pathological activation of the antigen‐specific immune system and exacerbating inflammatory bowel disease and allergic symptoms.

This study examines the role of the salivary glands in oral homeostasis by analyzing changes in oral microflora and salivary sIgA upon the induction of local inflammation in periodontal tissues in a mouse model of experimental periodontitis.

## 2 Materials and Methods

### 2.1 Mice

Four‐week‐old female BALB/c mice were purchased from CLEA Japan Inc. (Tokyo, Japan). The mice were bred in conventional facilities and fed regular chow and water until the experiment. All of the mice used in this study were maintained at the animal center of Showa University. After being allowed to acclimate to the conditions for 4 weeks, the mice were used in the experiment (at 8 weeks old). The study design was approved by the Ethics Committee for Animal Experiments of Showa University (approval numbers: 15050, 224025) and conducted in accordance with the Guidelines for Animal Experiments of Showa University.

### 2.2 Experimental Periodontitis Model

Murine periodontitis was induced by ligature placement, following the protocol reported by Marchesan et al. [20]. The mice were randomly assigned to the following three groups: (i) control group (Ctrl), (ii) ligation for 1 week group (L1w), and (iii) ligation for 2 weeks group (L2w). The mice were anesthetized intraperitoneally with 0.75 mg/kg medetomidine (Nippon Zenyaku Kogyo, Fukushima, Japan), 4.0 mg/kg midazolam (Sandoz K.K., Tokyo, Japan), and 5.0 mg/kg butorphanol (Meiji Animal Health, Kumamoto, Japan). Then, 4‐0 silk sutures (Bearmedic, Ibaraki, Japan) were inserted between the maxillary first and second molars on each side separately. To prevent loss of the ligature, the buccal and oral ends of the suture were tied off. Mice of the control group were only anesthetized. After ligature placement, 0.75 mg/kg atipamezole (Nippon Zenyaku Kogyo) was administered intraperitoneally as a medetomidine antagonist. Ctrl group was also administered at the same time. The silk sutures remained in place for 7 and 14 days. The mice were euthanized at the end of each experiment.

### 2.3 Cohousing Experiment

In the cohousing experiment, the mice were randomly assigned to two groups: (i) non‐cohoused control group (NCo) and (ii) recipient mouse group (Rcp). The experimental periodontitis model mice were placed in the same cage as the untreated mice and allowed to cohouse for 1 week. Mice were euthanized at the end of each experiment.

### 2.4 Histological Analysis

For histological analysis, the salivary glands and maxillary bone were removed from the mice. The salivary glands were placed in Optimal Cutting Temperature compound (Sakura Finetek, Tokyo, Japan). The maxillary bone was fixed with 4% phosphate‐buffered formaldehyde (PFA) (Muto Pure Chemicals, Tokyo, Japan) overnight and demineralized with 10% EDTA (Dojindo, Kumamoto, Japan) for 1 month, followed by embedding in paraffin. All sections were sliced at a thickness of 5 µm. Histological examination was performed using the sections stained with hematoxylin and eosin (Sakura Finetek).

### 2.5 Micro‐Computed Tomography Analysis

After removal of the mouse heads, the skin and mandible were removed and fixed with 80% ethanol. The mandible bones were scanned at 80 kV using a ScanXmate‐L090H (Comscantecno, Yokohama, Japan). Images were reconstructed using the TRI/3D‐BON‐FCS system (Ratoc System Engineering, Tokyo, Japan). The alveolar bone area was measured using the ImageJ software package (National Institutes of Health, Bethesda, MD, USA) to enclose the area from the proximal maxillary first molar to the distal maxillary second molar.

### 2.6 Immunofluorescence Staining

Frozen salivary gland sections were dried, treated with 4% PFA, and then blocked with 5% donkey serum (Jackson ImmunoResearch Laboratories, West Grove, PA, USA)/0.3% Triton X‐100 (Wako Pure Chemical Industries, Osaka, Japan) in phosphate‐buffered saline (PBS). The sections were incubated with anti‐CD45 rat mAb (30‐F11) (BioLegend, San Diego, CA, USA) and anti‐EpCAM Rabbit mAb (D9S3P) (Cell Signaling Technology, Danvers, MA, USA). Then, Alexa Fluor 647‐conjugated donkey anti‐rabbit IgG (H + L) (Jackson ImmunoResearch Laboratories) and Cy3‐conjugated donkey anti‐rat IgG (Jackson ImmunoResearch Laboratories) were applied. Finally, the sections were incubated with DAPI for nuclear staining and examined with an inverted laser microscope (Observer.Z1; ZEISS, Baden‐Württemberg, Germany). The number of positive cells per cm² was determined manually.

### 2.7 Immunohistochemical Staining

Frozen salivary gland sections were dried and treated with 4% PFA. Then, 10 mM sodium citrate buffer (pH 6.0) was added and antigens were retrieved. Subsequently, the sections were treated with endogenous peroxidase with the addition of 3% H₂O₂/methanol, followed by blocking with 5% goat serum/0.3% Triton X‐100 in Tris‐buffered saline (TBS). Incubation of the sections with primary antibody FITC‐conjugated anti‐mouse IgA (C10‐3) (BD Biosciences, Franklin Lakes, NJ, USA) was then carried out, after which goat anti‐rabbit IgG, HRP‐linked antibody (Santa Cruz Biotechnology, Dallas, TX, USA) was applied. Finally, protein detection was performed with the DAB substrate (K3468) (DAKO, Santa Clara, CA, USA).

### 2.8 Flow Cytometric Analysis

Staining of IgA⁺ cells were performed with the following antibodies: APC Cy7‐conjugated anti‐CD45.2 (Ly5.2) (104) (BioLegend) and APC‐conjugated anti‐IgA (11‐44‐2) (SouthernBiotech, Birmingham, AL, USA). Staining of macrophages, neutrophils, and dendritic cells was performed as follows: FITC‐anti‐mouse F4/80(BM8) (BioLegend), PE‐anti‐mouse/human CD11b(M1/70) (BioLegend), PerCP‐anti‐mouse Ly6C(Hk1.4) (eBioscience, San Diego, CA, USA), APC‐anti‐mouse Ly‐6G/Ly‐6C(Gr‐1) (RB6‐8C5) (BioLegend), APC Cy7‐conjugated anti‐CD45.2 (Ly5.2), and Brilliant Violet 421‐anti‐mouse CD11c (N418) (BioLegend). Anti‐CD45.2 (Ly5.2) was used as an antibody against CD45 in this study, serving as a marker for a pan‐leukocyte antigen. Flow cytometry was performed using a FACSVerse flow cytometer (BD Biosciences) and analyzed using FlowJo software (BD Biosciences).

### 2.9 ELISA

After the intraperitoneal administration of mixed anesthetics to mice, 0.02% pilocarpine (Santen, Osaka, Japan) was administered intraperitoneally to stimulate salivation. Saliva was then collected from the mouth for 10 min. The sample was centrifuged and the obtained supernatant was collected. Meanwhile, the mice were euthanized using carbon dioxide, and their peripheral blood was collected by puncturing the heart. The serum was coagulated and collected by centrifugation. To measure the amounts of IgA in the saliva and serum samples obtained as described above, ELISA was performed using IgA mouse Uncoated ELISA Kit (88‐50450‐88; Invitrogen, Waltham, MA, USA).

### 2.10 Isolation of Immune Cells From the Salivary Glands and Cervical Lymph Nodes

For immune cell isolation, cervical lymph nodes were placed in Dulbecco's modified Eagle's medium (DMEM) containing 2% fetal bovine serum (FBS), homogenized using a glass tube, and filtered through 150 μm metal filters. In addition, the salivary glands were homogenized on glass slides and placed in DMEM containing 10% FBS. The sample was occasionally mixed every 10 min a total of four times at 37°C. For homogenization, 0.5 mg/mL collagenase (Roche, Basel, Switzerland), 1 mg/mL hyaluronidase (Sigma‐Aldrich, St. Louis, MO, USA), and 10 μg/mL DNase (Roche) were used. Next, 5 mM EDTA/DMEM was added and filtered through a 150 µm metal filter. The samples were then centrifuged and washed twice with 10% FBS/DMEM. Finally, they were filtered through a 40 µm nylon mesh.

### 2.11 The 16S rRNA Amplicon Sequencing Analysis

The oral cavity of each mouse was washed with 500 µL of sterile PBS and the wash was collected. Bacterial flora analysis was performed by Bioengineering Lab. Co. (Kanagawa, Japan). After adding Lysis Solution F (Nippon Gene, Tokyo, Japan) to the samples, they were ground at 1500 rpm for 2 min using a Shake Master Neo (Bio‐Medical Science, Tokyo, Japan). The samples were then centrifuged and DNA was purified from the supernatant using Lab‐Aid824s DNA Extraction kit (ZEESAN, Xiamen, China). Synergy LZ (Agilent Technologies, Tokyo, Japan) and QuantiFlour dsDNA System (Promega, Tokyo, Japan) were used to measure the concentration of the DNA solution. Libraries were prepared using a two‐step tailed PCR method. Sequencing was performed using the MiSeq system and the MiSeq Reagent Kit v3 (Illumina, San Diego, CA, USA) at 2×300 bp. Sequences were carefully selected using the fastx_barcode_splitter tool in the FASTX‐toolkit (ver. 0.0.14). Primer sequences were removed from the extracted reads using fastx_trimer of the FASTX‐toolkit. Sequences with quality values of less than 20 were then removed using sickle (ver. 1.33), and sequences of less than 130 bases in length and their paired sequences were discarded. Reads were joined using the paired‐end read joining script FLASH (ver. 1.2.11). After removing chimeric and noise sequences with the dada2 plugin of Qiime2 (ver. 2023.2), representative sequences and ASV tables were output. Phylogeny was estimated by comparing the obtained representative sequence with the 97% OTU of Greengene (ver. 13_8). Chloroplast and *Rickettsia*‐derived mitochondrial reads were excluded. Alignment and phylogeny plug‐ins were used to construct the phylogenetic tree. Analysis of alpha and beta diversity was performed using the diversity plugin of Qiime2. LEfSE (ver. 1.0.8) was used to test whether there were strains with different relative abundances between groups. The reported sequence data are available in the DDBJ database under accession number PRJDB18706.

### 2.12 RNA Preparation

The salivary glands were removed from mice and placed in RNA Later (Invitrogen) overnight. After adding buffer RLT (Qiagen, Hilden, Germany) to the samples, they were ground at 1500 rpm for 3 min using a Shake Master Neo. RNA was then extracted using the RNeasy Mini Kit (Qiagen).

### 2.13 Gene Expression Analysis

The RNA sequencing was performed by Rhelixa (Tokyo, Japan). The quality scores of the sequence reads were evaluated using FastQC (Version 0.11.7). Low‐quality (< 20) base and adapter sequences were trimmed using Trimmomatic software (Version 0.38) with the following parameters: ILLUMINACLIP:path/to/adapter.fa:2:30:10, LEADING:20, TRAILING:20, SLIDINGWINDOW:4:15, MINILEN:36. Trimmed reads were mapped to the reference genome using HISAT2 (Version 2.1.0). The number of reads mapped to known exon regions was estimated using featureCounts (Version 1.6.3), and read counts were normalized to transcripts per million (TPM). Samples were clustered using the Wald method based on the Euclidean distance of normalized counts, with the analysis conducted using the stats (Version 3.6.1) and gplots (Version 3.0.1.1) R packages. Principal component analysis (PCA) was performed on the normalized counts, projecting each sample onto a two‐dimensional plane defined by the first and second PCA axes. Pearson's correlation coefficients for normalized counts were calculated to assess sample correlations. Histograms and paired plots, as well as heat maps based on z‐scores of normalized counts, were generated using the stats and gplots R packages. Raw read counts were normalized using relative log normalization (RLE) and differential expression analysis was performed using DESeq. 2 (Version 1.24.0). The Benjamini–Hochberg (BH) method was used to detect differentially expressed genes (DEGs) with thresholds of |log2FC (fold change) | > 1 and adjusted *p*‐value < 0.05. Heat maps and volcano plots were compared between mutant genes using the RIAS Omics Analysis System (Rhelixa). GOATOOLS (Version 1.1.6) was used for gene ontology (GO) enrichment analysis of DEGs. The reported sequence data are available in the DDBJ database under accession number PRJDB18749.

### 2.14 Statistical Analysis

The significance of differences was calculated by Student's unpaired *t*‐test. Values of *p* < 0.05 were considered statistically significant.

## 3 Results

### 3.1 Induction of Localized Inflammation Experimental Periodontitis in Mice

**Figure 1:**
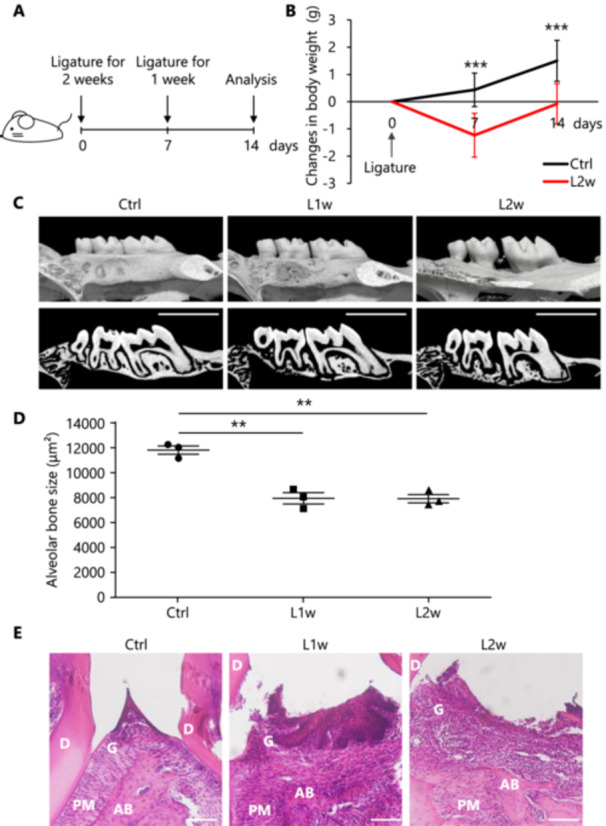
Induction of experimental periodontitis in mice and histological analysis of periodontal tissues. (A) Experimental periodontitis model mice; 5‐0 silk sutures were ligated between the maxillary first and second molars of BALB/c 8‐week‐old female mice and analyzed 1 (L1w) or 2 weeks later (L2w) for comparison with ligature control mice (Ctrl). (B) Mice were weighed on Days 7 and 14, with Day 0 being the day of ligaturing (Ctrl, *n* = 9; L2w, *n* = 9). (C) Micro‐CT images of maxillary bones in three groups (Ctrl, L1w, L2w) of mice. The upper panel shows a 3D image and the lower panel shows a sagittal plane image (scale: 200 μm). (D) The alveolar bone area from the mesial root of the maxillary first molar to the distal root of the maxillary second molar (Supporting Information S1: Supplemental Figure 1B red frame) was measured using ImageJ from the μCT sagittal plane image shown in (C) (Ctrl, *n* = 3; L1w, *n* = 3; L2w, *n* = 3). (E) HE staining images of maxillary bones in each group of mice. Magnification is 20× (scale: 100 μm); D (dentin), AB (alveolar bone), PM (periodontal membrane), G (gingiva). Data are expressed as mean ± SD. Statistical significance was determined using a two‐tailed unpaired *t*‐test. Statistical significance is indicated as follows: **p < 0.01, ***p < 0.001.

As a model for local inflammation in the oral cavity, experimental periodontitis model mice were generated by inserting silk threads into the cervical region between the adjacent first and second molars (Supporting Information S1: Supplemental Figure 1A). Body weight changes were monitored 1 and 2 weeks after initiation, after which oral tissues were harvested and compared with those in the control group (**Ctrl**) for histological changes in periodontal tissue upon the induction of inflammation (Figure 1A). The treated mice lost about 5% of their body weight after 1 week (**L1w**), but recovered to the pre‐treatment level after 2 weeks (**L2w**) (Figure 1B). The pathological changes in the periapical tissues of the ligature‐treated teeth were confirmed by micro‐computed tomography (micro‐CT). The alveolar bone in the interdental area showed an irregular and discontinuous appearance, and mild alveolar bone resorption was observed. After 2 weeks of ligature treatment, more significant alveolar bone resorption was observed in the interdental and root bifurcation areas (Figure 1C). Bone volume changes in the alveolar bone trabeculae in the region of the first and second molars were compared by micro‐CT (Figure 1D, highlighted in red in Supporting Information S1: Supplemental Figure 1B). The results showed that significant resorption developed at 1 and 2 weeks after ligature treatment compared with the findings in the control. Then, the tissue specimens were examined by HE staining to determine the degree of inflammation induced in the periodontal tissues. The region between the first and second molars with the ligature inserted is shown in Figure 1E. One week after the ligature procedure, thickening of the gingival keratinizing epithelium between the molars due to the pressure contact of the threads and the presence of fibroblasts in the stromal tissue were observed (middle panel, Figure 1E). Two weeks later, disruption of gingival epithelial continuity, disruption of keratinized epithelium, epithelial ulceration, and infiltration of numerous inflammatory cells in the stromal tissue were observed (right panel, Figure 1E). In control mice, the junctional epithelium in the area along the crown and root of the tooth was entirely preserved (left panel, Figure 1E). Previous study reports showed that alveolar bone resorption and other symptoms were observed after 5 days of treatment, which was consistent with our results [21]. These results confirm that, in our experimental periodontitis model mice, induced inflammation is localized in the periodontium in the oral cavity.

### 3.2 Effects of Induced Inflammation of Periodontal Tissue on Salivary Glands

**Figure 2:**
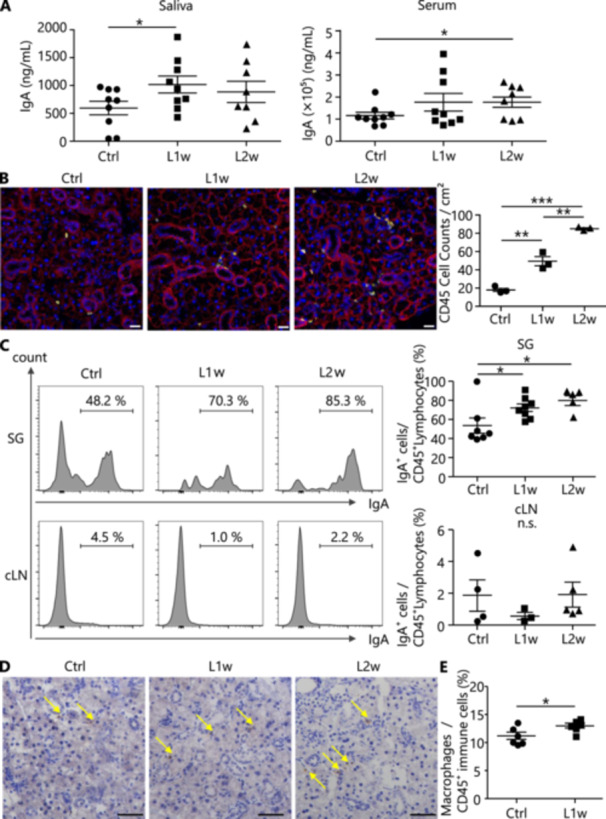
Histological changes in salivary glands associated with the development of experimental periodontitis. (A) IgA levels in saliva and serum were measured using ELISA. Saliva (Ctrl, *n* = 9; L1w, *n* = 9; L2w, *n* = 8), serum (Ctrl, L1w, L2w, *n* = 9). (B) Immunofluorescence staining images in the submandibular gland of each mouse. Yellow indicates CD45, red indicates EpCAM, and blue indicates DAPI. Magnification is 40× (scale: 20 μm). (C) The proportions of IgA⁺ cells among CD45⁺ cells in salivary glands (SG) and cervical lymph nodes (cLN) of each mouse using flow cytometry. SG (Ctrl, *n* = 7; L1w, *n* = 8; L2w, *n* = 4), cLN (Ctrl, *n* = 4; L1w, *n* = 3; L2w, *n* = 5). (D) Immunohistochemical staining images in the submandibular gland of each mouse. Brown indicates staining with IgA. Magnification is 40× (scale: 50 μm). (E) The proportions of macrophages among CD45^+^ cells in the salivary glands of each mouse using flow cytometry (Ctrl, *n* = 6; L1w, *n* = 6). Macrophages were gated with CD45^+^Gr‐1^−^F4/80^+^CD11b^+^. The experiment was repeated twice. Data are expressed as mean ± SD. Statistical significance was determined using a two‐tailed unpaired *t*‐test. Statistical significance is indicated as follows: *p < 0.05, **p < 0.01, ***p < 0.001, ns: *p* > 0.05.

Next, we examined whether the induction of inflammation in localized periodontal tissues influenced the salivary glands, particularly their production of IgA, an important immunoglobulin in the oral mucosa. Saliva was collected from the oral cavity of experimental periodontitis model mice 1 and 2 weeks after treatment, and concentrations of salivary IgA were measured. The results showed that, after 1 week of treatment, salivary IgA levels increased significantly, and serum IgA showed a similar tendency to increase. Moreover, after 2 weeks, serum IgA increased significantly, and salivary IgA also tended to increase (Figure 2A). It was thought that the increased IgA was triggered by local inflammation induced in the periodontal tissues. Histological analysis of the submandibular glands showed no histopathological findings such as inflammation, atrophy of the adenocytes, or fibrosis (Supporting Information S1: Supplemental Figure 2A). The accumulation of immune cells in the salivary glands after ligature treatment, represented by the pan‐immune cell marker CD45, was increased after 1 and 2 weeks (Figure 2B). Therefore, to investigate immunological changes in the salivary glands of experimental periodontitis model mice, the salivary glands and cervical lymph nodes were analyzed for changes in the proportion of IgA⁺ cells. The proportion of IgA⁺ cells among pan‐immune cells (CD45⁺) in the salivary glands after treatment was compared with that in the control group by flow cytometry (Supporting Information S1: Supplemental Figure 2B). Histogram analysis showed that the proportion of IgA⁺ cells in the salivary glands was significantly increased at both 1 and 2 weeks compared with that in control mice (Figure 2C). Additionally, a time‐course analysis was conducted at 3, 5, and 7 days after induction of experimental periodontitis, showing that the number of IgA^+^ cells peaked around day 5 and stabilized by 1 week (data not shown). Similarly, the percentage of macrophages/pan‐immune cells was significantly higher at L1w (Figure 2E). An elevation in dendritic cells was also observed, while neutrophil levels showed no significant change (Supporting Information S1: Supplemental Figure 2D,E). In contrast, the proportion of IgA⁺ cells in the cervical lymph nodes did not change during either period. These findings suggest that local oral inflammation has a greater effect on the production of antibodies released into saliva. More detailed histological observations of the salivary glands showed an increase in IgA⁺ cells in the salivary glands of the experimental periodontitis model mice. In L1w and L2w mice, IgA^+^ cells accumulated in the intercellular spaces between glandular cells and around ducts, whereas in control mice, they were scattered in the intercellular spaces (Figure 2D). In contrast, the number of IgA^+^ cells in the lamina propria layer of the large intestine did not significantly increase after either 1 or 2 weeks, indicating that the impact of oral inflammation on remote tissues such as the intestinal tract was limited (Supporting Information S1: Supplemental Figure 2C). These results confirm that the induction of inflammation in periodontal tissues via the ligature procedure effectively increases IgA antibody‐producing cells, particularly in the salivary glands, thereby promoting IgA secretion in saliva.

### 3.3 Effects of Altered Salivary Gland Function on the Oral Microflora in the Experimental Periodontitis Model

**Figure 3:**
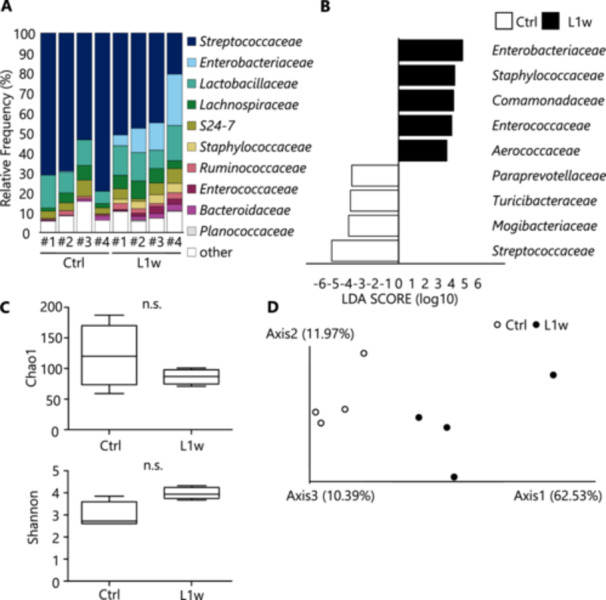
Effects on oral microbiome composition following induction of experimental periodontitis in mice. Microbiome analysis using 16S rRNA amplicon sequencing was performed using oral rinses from Ctrl and L1w group mice. (A) The microflora in the Ctrl and L1w groups at the family level. (B) The bacterial families that significantly increased in the Ctrl or L1w group. (C, D) Alpha diversity (C) and beta diversity (D) in the oral microflora of the Ctrl and L1w groups. Alpha diversity was assessed using Chao1 and Shannon indices to evaluate the richness and evenness of the microbial communities. Data are expressed as mean ± SD. Statistical significance was determined using a two‐tailed unpaired *t*‐test. Statistical significance is indicated as follows: ns: *p* > 0.05. Data are representative of two independent experiments.

Metagenomic analysis using 16S rRNA amplicon sequencing was used to compare the experimental periodontitis model and control mice to investigate the impact of local oral inflammation on commensal microflora. In the analysis at the family level, *Streptococcaceae*, *Lactobacillaceae*, *Lachnospiraceae*, *S24‐7*, and *Ruminiciccaceae* were the major oral microflora in control mice, whereas in experimental periodontitis model mice, *Enterobacteriaceae*, *Lactobacillaceae*, *Lachnospiraceae*, *S24‐7*, and *Ruminiciccaceae* were the major microflora. The findings showed that experimental periodontitis led to the addition of *Enterobacteriaceae*, *Staphylococcaceae, Enterococcaceae*, and *Bacteroidaceae* as major taxa within the oral microflora (Figure 3A). Next, to analyze significant changes at the family level, linear discriminant analysis effect size (LEfSe) analysis was performed to compare the characteristics of the oral microflora of experimental periodontitis model and control mice (Figure 3B). On the basis of a comprehensive analysis of two independent experiments, *Staphylococcaceae* and *Enterococcaceae* were dominant in experimental periodontitis model mice, with a relative decrease in *Streptococcaceae*. A decreasing trend in alpha diversity was observed in L1w (Figure 3C), while beta‐diversity did not differ significantly between the oral microflora of ligature and control mice (Figure 3D). In addition, 16S rRNA amplicon sequencing analysis of the intestinal microflora showed increases of *Staphylococcus* and *Enterococcus*, indicating that oral ligature treatment induces microbial alteration in the intestinal tract (Supporting Information S1: Supplemental Figure 3A–D). Taking these findings together, the ligature treatment generally induced dysbiotic constitutive changes in the commensal microflora of the mice.

### 3.4 Gene Expression Analysis in Salivary Gland Tissues of Experimental Periodontitis Model Mice

**Figure 4:**
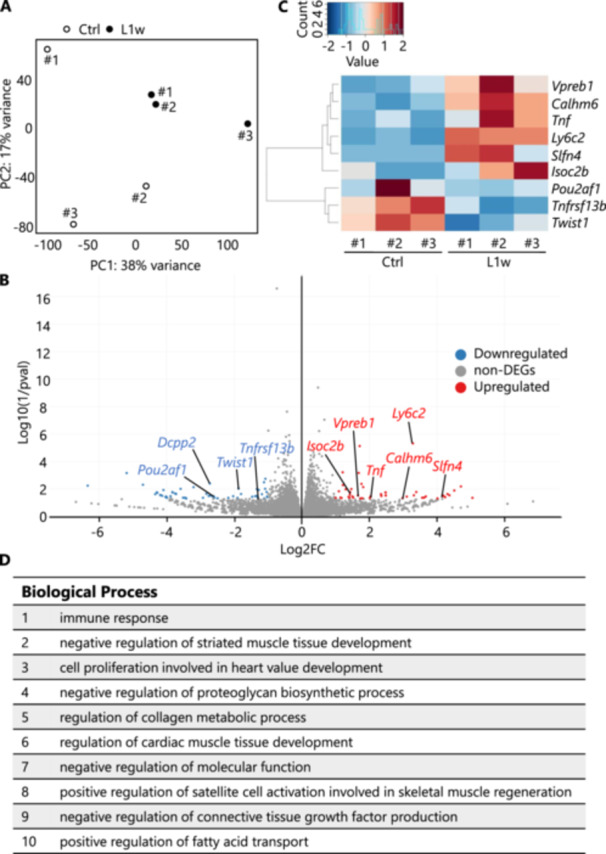
Differential gene expression in salivary gland tissues of mice with ligature‐induced experimental periodontitis. Salivary glands were removed from mice in the Ctrl and L1w groups, RNA was isolated, and RNA sequencing was performed. (A) A volcano plot showing the differentially expressed genes (DEGs) between the Ctrl and L1w groups. (B) Principal component analysis (PCA) between Ctrl and L1w group samples. (C) Heat map showing DEGs between Ctrl and L1w groups. (D) Gene ontology analysis.

To gain more detailed insight into the effects of local inflammation in periodontal tissues on salivary glands, we analyzed changes in gene expression in salivary gland tissue 1 week after ligature treatment by bulk RNA sequencing. PCA of gene expression in the salivary glands of experimental periodontitis model and control mice showed convergence to two groups (Figure 4A). As for individual genes, B cell‐associated genes, for example, that promote B‐cell maturation and plasma cell differentiation (*Tnfrsf13b* and *Pou2af1*) were downregulated, whereas a gene considered to be expressed in immature B cells (*Vpreb1*) showed elevated expression. Furthermore, the expression of macrophage‐related genes such as inflammatory cytokines (*Tnf*) and genes associated with monocyte and macrophage activation (*Ly6c2*, *Slfn4*, *Calhm6*) was detected. The expression of *Twist1*, which is associated with TNF‐α, was decreased, while *Isoc2b*, a hydrolytic enzyme that negatively regulates P16 INK4a (a molecule that inhibits cell division), exhibited increased expression. Furthermore, the expression of the *Dcpp2* gene, which is involved in nutrient intake, was also decreased (Figure 4A–C, Supporting Information S1: Supplemental Figure 4A,B, Supplementary Tables 1 and 2). In general, gene expression in the salivary glands of experimental periodontitis model mice showed a tendency to suppress B‐cell activation and to activate macrophage/monocyte activation. GO analysis showed elevations in “immune response,” “negative regulation of striated muscle tissue development,” and “cell proliferation involved in heart valve development” (Figure 4D). Other elevations were observed in terms related to “proteoglycans,” “collagen metabolism,” “connective tissue,” and “adipose tissue.” In summary, the localized inflammation induced in the periodontal tissue appeared to extend to the perioral region in 1 week, causing changes associated with the immune system, including plasma cells, B cells, macrophages, and monocytes in the salivary glands.

### 3.5 Alteration of Oral Microbiota and Salivary IgA in Recipient Mice Using a Cohousing Model

**Figure 5:**
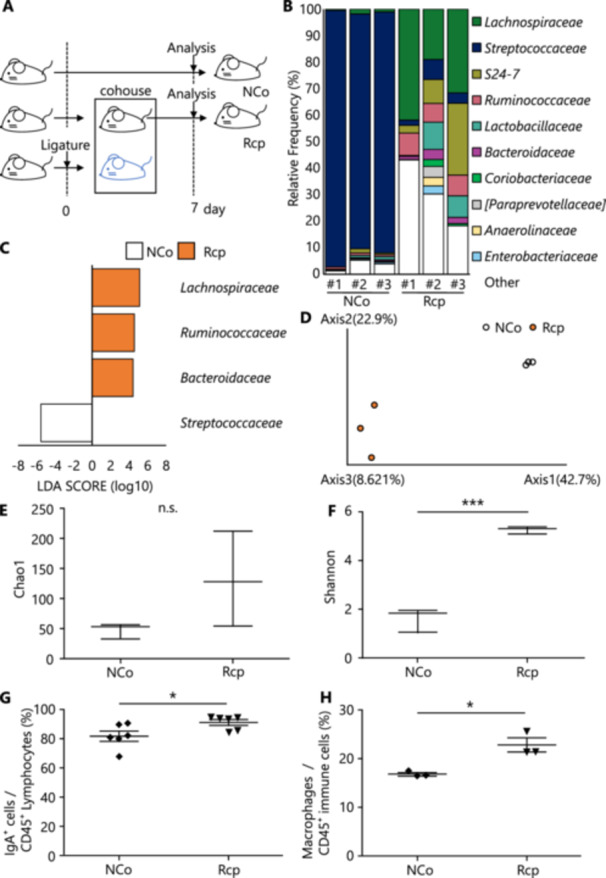
Effects in the oral microbiota of recipient mice and the salivary glands in experiments following cohousing with experimental periodontitis model. (A) Experimental periodontitis model mice were prepared in the same manner as in Figure 1A, cohoused with untreated mice, and analyzed 1 week later. We compared non‐cohabiting control mice (NCo) with recipient mice (Rcp) to examine the alteration of the oral microbiota. (B) The microflora in the NCo and Rcp groups at the family level. (C) The bacterial families that significantly increased in the NCo or Rcp group. (E–F) Alpha diversity (E, F) and beta diversity (D) in the oral microflora of NCo and Rcp groups. Alpha diversity was assessed using Chao1 and Shannon indices to evaluate the richness and evenness of the microbial communities. (G, H) The proportions of IgA⁺ cells (G) and macrophages (H) among CD45⁺ cells in the salivary glands of each mouse using flow cytometry: IgA⁺ cells (NCo, *n* = 6; Rcp, *n* = 6), macrophages (NCo, *n* = 3; Rcp, *n* = 3). Macrophages were gated with CD45⁺Gr‐1‐F4/80⁺CD11b⁺. Data are expressed as mean ± SD. Statistical significance was determined using a two‐tailed unpaired *t*‐test. Statistical significance is indicated as follows: *p < 0.05, ***p < 0.001, ns: *p* > 0.05.

Finally, to determine whether alteration of the oral microbiota affects the salivary glands, we cohoused mice to transfer the oral microflora between them and then examined the effects on the salivary glands. The experimental procedure involved a cohousing model in which mice with periodontitis induced by ligation as donors of oral microflora cohabited with normal mice as recipients for 7 days (Figure 5A). Our analysis of the composition of the oral microflora showed significant changes in the recipient mice (**Rcp**) compared with the findings in non‐cohabiting control mice (**NCo**) (Figure 5B). The relative abundance of obligate anaerobes such as *Lachnospiraceae*, *Ruminococcaceae*, and *Bacteroidaceae* increased in the recipient mouse group, while that of the endogenous oral *Streptococcaceae* decreased (Figure 5C). Microbial diversity analysis showed a distinct microbial separation between Rcp and NCo groups (Figure 5D–F). To assess the impact of oral microbiota and the immune response of the salivary glands, we analyzed the immune systems: the percentage of IgA⁺/pan‐immune cells was significantly higher in the Rcp group (*p* < 0.05, Figure 5G). Similarly, the percentage of macrophages/pan‐immune cells was significantly higher in the Rcp group (*p* < 0.05, Figure 5H). There was also an elevation in dendritic cells, while neutrophils showed no particular change (Supporting Information S1: Supplemental Figure 5A,B). Otherwise, no histological changes were observed in the periodontal tissues and salivary glands of the Rcp group (Supporting Information S1: Supplemental Figure 5C–E). These results indicate that the oral microbiota is transmitted between individual mice, resulting in changes in the microbiota and the community composition in the recipient mice. Furthermore, alterations in the oral microbiota cause significant changes in the local immune response in the salivary glands. This suggests a significant association between oral microbial infection and modulation of the host immune system in the salivary glands.

## 4 Discussion

In this study, we demonstrated that localized periodontitis induces IgA production in salivary glands by altering the oral microbiome. Previous studies on animal models of ligature treatment showed that the induction of inflammatory cytokines in the immune cells of the surrounding periodontal and mucosal tissues after ligature application causes periapical bone destruction through various mechanisms [22, 23, 24, 25]. Our finding has significant implications for our understanding of the relationship between oral health and the immune system. In recent years, research has progressed on the impact of oral conditions on the whole body. In particular, regarding intestinal inflammation such as inflammatory bowel disease and Crohn's disease, it has been shown that the mechanism of disease pathogenesis via interaction between the oral cavity and intestinal tract involves activated T cells derived from the oral cavity migrating to the gastrointestinal mucosa by ligature treatment and exacerbating the symptoms of enteritis [17, 26]. While salivary gland tissues are closely related to the oral cavity, the extent to which inflammation of periodontal tissues and other pathological conditions affect salivary gland tissues has yet to be thoroughly investigated. Only limited reports on the effects of ligature treatment on salivary gland tissues have been published. In animal models, it is reported that ligature treatment in rats increases TNF‐α receptor expression and induces apoptosis in salivary glands in response to elevated serum TNF‐α levels [13]. A previous study examining saliva from human gingivitis patients reported increased IgA secretion from the parotid gland in experimentally induced human gingivitis without oral hygiene [27]. Meanwhile, it has been reported that IgA secretion is decreased in patients with invasive periodontitis [28]. Thus, the results regarding the increase or decrease in IgA secretion in oral inflammation are not consistent and may be influenced by differences in saliva collection methods and other factors. In this study, we observed increased IgA‐producing cells in salivary gland tissue. We believe that inflammation in the periodontal tissues induces IgA secretion from the salivary glands and that the alteration of the bacterial flora is involved in this mechanism. Because inflammation in the periodontal tissues promotes the production of antimicrobial peptides, cytokines, chemokines, and other substances from the surrounding tissues [29], once the balance of the oral flora is disrupted, this may trigger the accumulation of distal IgA‐secreting cells at the salivary glands.

Although the specific mechanism by which oral dysbiosis induces IgA production from salivary glands has not been clarified, two main mechanisms can be assumed from previous studies: (1) IgA‐producing cells such as those in the intestinal tract migrate to salivary gland tissues and secrete sIgA into saliva, and (2) macrophages in salivary gland tissues take up salivary duct‐derived bacterial antigens and plasma cells secrete the corresponding antibodies into saliva. In the present study, IgA‐producing cells in the intestinal tract did not show significant quantitative changes, and the bulk RNA sequencing results suggest rather that dysbiosis of the oral flora may allow bacterial antigens to enter the salivary ducts and activate macrophages in the salivary glands to induce IgA⁺ cells [30, 31, 32].

In this study, increases in *Staphylococcus* and *Enterococcus* were observed under conditions of oral dysbiosis. It has recently been revealed that these bacterial species are associated with periodontal disease and oral dysbiosis. For example, in a previous study [33], among the bacterial species newly identified as being highly associated with periodontal disease were *Enterococcus* species such as *Enterococcus faecalis* and *Staphylococcus warneri*. It is notable that the inclusion of *Staphylococcus* species is consistent with the composition of the oral microbiota observed after the induction of periodontitis with ligatures in this study. The antimicrobial resistance and virulence factors of *Staphylococcus* species may be involved in periodontal disease. An increased proportion of *Staphylococcus* species in subgingival plaque may be associated with the severity of advanced periodontal disease [34]. Further detailed experiments are needed to determine whether these bacteria are the cause or the consequence of the induction of dysbiosis in the oral microflora.

Our research on the dysbiosis induced in the intestinal tract by the oral ligature treatment (Supporting Information S1: Supplemental Figures 2C and 3A–D) has the potential to significantly impact our understanding of intestinal dysbiosis and mucosal antibody production. We found no increase in IgA production in the intestinal tract, suggesting no change in antibody production in the intestinal mucosa. Instead of an increase in mucosal antibodies, our data suggest that intestinal dysbiosis may have been induced by the influx of ectopic bacteria into the intestinal tract due to dysbiosis in the oral cavity.

Results from previous studies suggested that disease‐inducing horizontal transmission of bacterial flora can occur between individuals when they are co‐housed, involving diseased and normal mice being kept in the same environment. In these studies, when mice possessing different bacterial flora were co‐housed together for 8 weeks, horizontal transmission of oral flora was less likely to occur if the microflora was stable [35, 36]. Meanwhile, changes in the bacterial flora associated with obesity or disease were horizontally transmitted to control mice cohoused with them within just 5 days [37]. Our study presents several important limitations that warrant careful consideration. The brief one‐week cohousing period may have resulted in acute bacterial flora changes with potentially heightened effects on host responses. While we observed alterations in salivary gland immune responses, these findings need validation through extended cohousing studies spanning beyond 1–2 weeks. The inherent coprophagic behavior of mice presents another significant consideration, as it may have induced temporary modifications in bacterial flora composition. Future experimental designs should incorporate strategies to minimize coprophagic behavior to enhance result reliability. Another crucial consideration is the methodological difference between the ligated and Rcp groups. The absence of ligatures in the Rcp group likely influenced bacterial adherence patterns [38], potentially explaining the disparities in bacterial flora profiles observed between Figures 3 and 5. While our approach aimed to examine salivary gland effects independent of ligature‐induced inflammation, our results should be interpreted as suggestive rather than conclusive evidence for horizontal transmission of oral flora. We acknowledge that various other factors may have influenced our observations and cannot be completely ruled out.

Gene expression analysis of ligature‐treated mice revealed changes in expression of several genes in salivary gland (Figure 4B,C). First, in genes related to B cells, the expression of *Pou2af1*, which promotes B‐cell maturation, and the BAFF receptor (*Tnfrsf13b*), a B‐cell‐activating receptor, was decreased, suggesting an increased proportion of immature B cells in the salivary glands [39, 40]. The expression of *Vpreb1*, a surface molecule of immature B cells, was also found to be increased [41]. These results suggest that plasma cell differentiation and activation may be suppressed in the salivary glands during the onset of periodontal disease. Among other upregulated genes, *Calhm6* is known to be expressed in macrophages and its product functions as a calcium ion channel, as well as being involved in promoting ATP release and synapse formation between macrophages and NK cells [42]. The *Slfn4* gene is also associated with macrophage activation functions because its expression is increased in activated macrophages [43]. Furthermore, *Twist1* is associated with TNF‐α and is involved in gene regulation via NF‐κB activity [44, 45]. Although its relationship with oral inflammation is unclear, we observed increased expression of a hydrolytic enzyme (encoded by the *Isoc2b* gene) that negatively regulates P16 INK4a, a molecule that inhibits the transition from the G_1_ to the S phase of the cell cycle and suppresses cell division [46]. There was also decreased expression of the *Dcpp2* gene, which is associated with nutrient intake [47]. These results suggest that local inflammation of periodontal tissue may affect IgA secretion from the salivary glands and induce tissue repair and remodeling.

The association between diseases of the salivary glands and oral microflora is an important area of research, and advancing our understanding of this topic is crucial. Sjögren's syndrome (SS) is a clinically important disease affecting the salivary glands. In addition to genetic factors, several environmental factors, such as infection with pathogenic microorganisms and smoking, may contribute to its development. Colony formation of the fungal species *Candida albicans* on the oral mucosa and an increase in *Lactobacillus* spp. on tooth surfaces have been observed in SS patients [48]. Furthermore, the presentation of antigens of specific bacteria such as *Prevotella melaninogenica* in the salivary glands of SS patients has been implicated in the pathogenesis of this disease [49]. The association between oral dysbiosis and salivary gland function observed in this study is consistent with findings from previous studies of SS. The increases in *Staphylococcus* and *Enterococcus* observed in the experimental periodontitis model (Figure 3A,B) are comparable to the changes in the bacterial flora seen in SS patients [48, 50]. In addition, the increased expression of macrophage‐related genes (*Ly6c2*, *Slfn4*, *Calhm6*) observed in this study suggests the activation of inflammatory and immune responses, a common feature of salivary gland changes associated with SS and aging. A previous study on gene expression in the salivary glands reported increased expression of chemokines involved in B‐cell migration in salivary gland epithelial cells from aged mice, and another group also reported increased expression of genes associated with B‐cell activation in salivary gland epithelial cells from patients with primary SS [51, 52]. The bulk RNA‐seq analysis in this study also showed changes in the expression pattern of B‐cell‐related genes, and further investigation of the relationship between oral dysbiosis induced in the experimental periodontitis model and salivary gland function may lead to the development of new therapeutic strategies for diseases involving the salivary glands such as SS.

This study has several limitations. First, surface markers such as Syndecan 1 (CD138) (positivity for which is an indicator of plasma cells) and CD19/CD80 (double positivity for which is an indicator of memory B cells) have not been analyzed. There was also insufficient information on the cellular function, including detailed phenotypic analysis of IgA⁺ cells in the salivary glands. Second, the antigen specificity of IgA to oral bacteria induced in experimental periodontitis model mice has not been studied. In addition, although inflammation was induced by ligature treatment in mice in this study, it is unknown whether it accurately reflects the oral condition of periodontitis patients.

The results of this study may be due to the effects of individual bacteria. Prospects for future work thus include analyses of the oral and intestinal immune responses in mouse models using the bacteria identified in this study, such as *Enterobacteriaceae*, *Staphylococcaceae*, *Enterococcaceae*, and *Bacteroidaceae*. Next, changes in inflammation and bacterial flora for longer than 2 weeks after ligature treatment could be investigated to obtain information on the mechanism behind the chronic progression of periodontitis. Using more detailed flow cytometric and immunohistochemical analyses, the process of recovery after ligature removal could be observed to understand the mechanism of recovery of oral microflora and immune response and to obtain useful insights regarding post‐treatment care. Furthermore, because bacterial short‐chain fatty acids such as acetic acid are known to induce IgA production, it is crucial to investigate the effect of acetic acid in saliva on IgA production and to determine the impact of these metabolites on immune responses, thereby exploring the possibility of new therapeutic approaches [53].

Our study demonstrated significant connections between localized periodontal inflammation, salivary gland function, and the oral microbiome. The experimental periodontitis model mice exhibited an increase in IgA‐producing B cells in the salivary glands, which was associated with alteration of the oral microbiome. Gene expression analysis further revealed that the immune response in the salivary glands was changed, with the downregulation of genes related to B‐cell maturation and the upregulation of those related to macrophage activation. Furthermore, our analyses of cohoused mice suggested that the alteration of the oral microbiome can induce similar immunological changes in the salivary grands of recipient mice. This highlights the potential for the oral microbiome to influence the host's immune environment beyond the immediate site of inflammation. Our findings emphasize the intricate interplay among oral health, salivary gland function, and systemic immunity, providing new insights into the mechanisms that maintain oral and overall health.

## Ethics Statement

The experimental protocol was approved by the Ethics Committee for Animal Experiments of Showa University (approval numbers: 15050, 224025). All animal experiments were conducted in accordance with the Guidelines for Animal Experiments of Showa University and the national guidelines for animal research in Japan. The study protocol was designed to minimize animal suffering and reduce the number of animals used.

## Conflicts of Interest

The authors declare no conflicts of interest.

1

The data that support the findings of this study are openly available in the DDBJ database under accession numbers PRJDB18706 and PRJDB18749.

## Supporting information

Supporting information.
