# Supplementary material for: Experimental Murine Periodontitis Increases Salivary Gland IgA‐Producing B Cells Following Oral Dysbiosis
Source: Microbiol Immunol. 2024 Dec 22;69(2):114–27. doi: 10.1111/1348-0421.13191 (PMC11789210; doi:10.1111/1348-0421.13191)
Supplement: Supplementary file 1 — Supporting information. [file MIM-69-114-s001.pptx]

## Slide 1
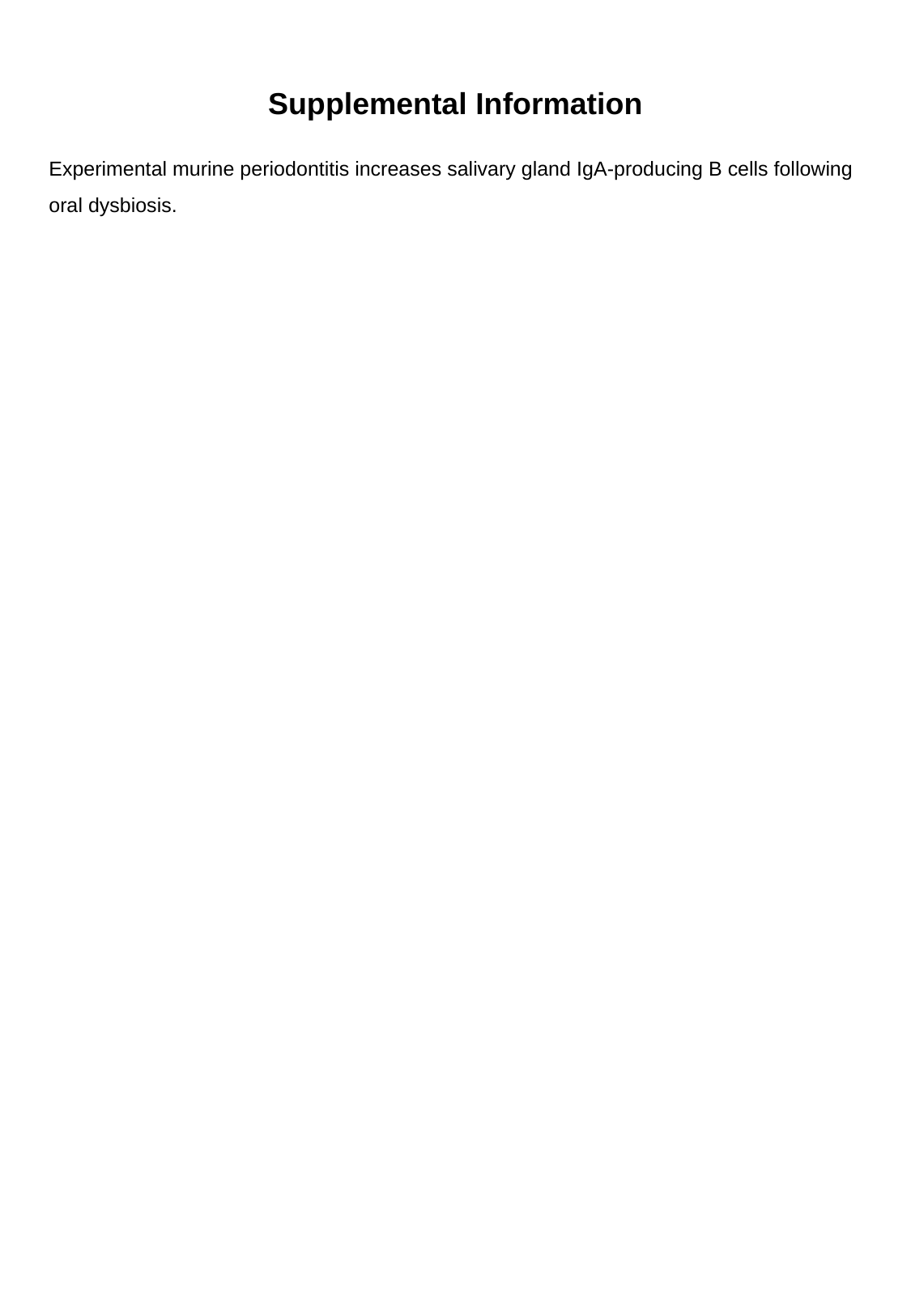

Supplemental Information
Experimental murine periodontitis increases salivary gland IgA-producing B cells following oral dysbiosis.

## Slide 2
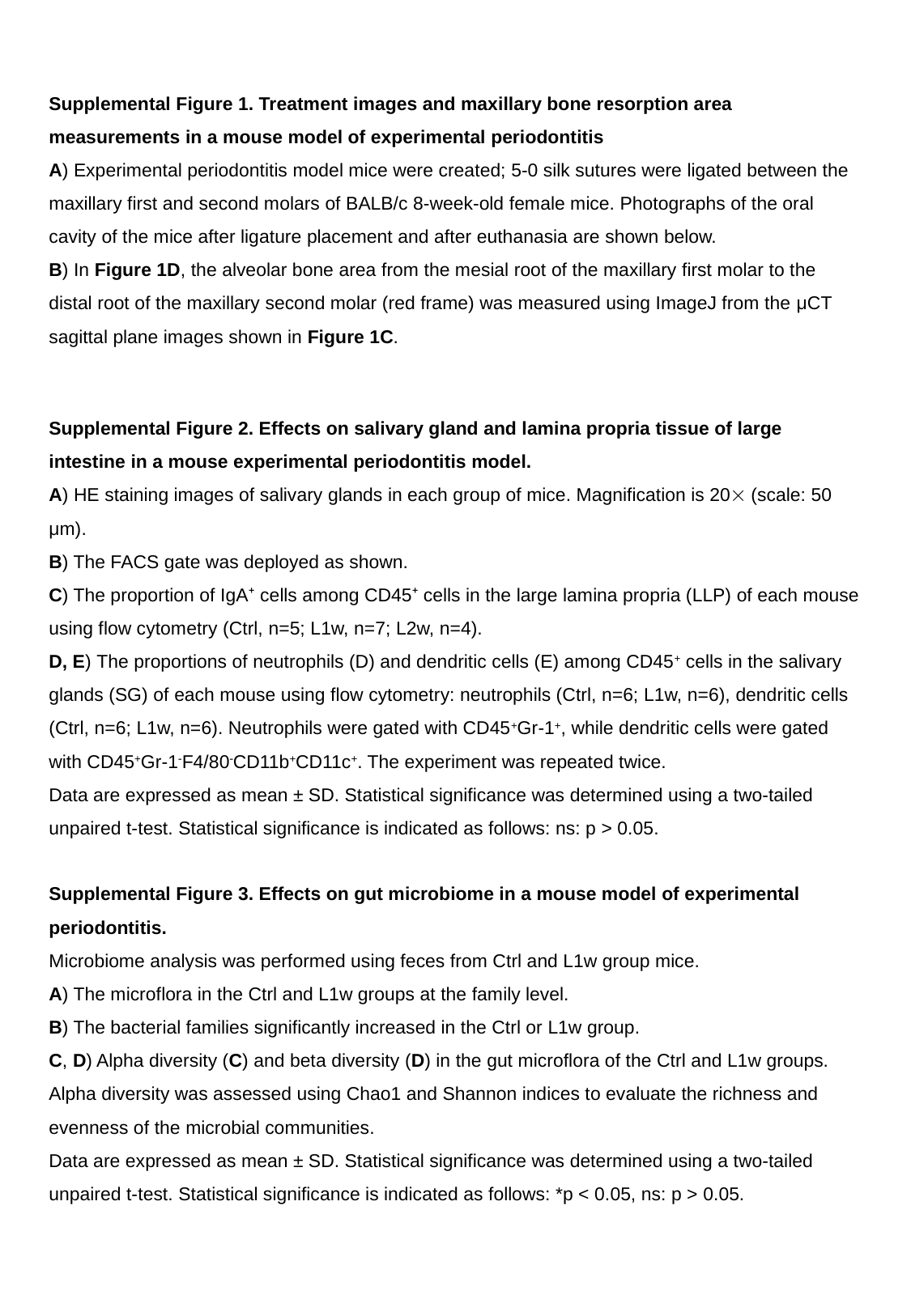

Supplemental Figure 1. Treatment images and maxillary bone resorption area measurements in a mouse model of experimental periodontitis
A) Experimental periodontitis model mice were created; 5-0 silk sutures were ligated between the maxillary first and second molars of BALB/c 8-week-old female mice. Photographs of the oral cavity of the mice after ligature placement and after euthanasia are shown below.
B) In Figure 1D, the alveolar bone area from the mesial root of the maxillary first molar to the distal root of the maxillary second molar (red frame) was measured using ImageJ from the μCT sagittal plane images shown in Figure 1C.
Supplemental Figure 2. Effects on salivary gland and lamina propria tissue of large intestine in a mouse experimental periodontitis model.
A) HE staining images of salivary glands in each group of mice. Magnification is 20× (scale: 50 μm).
B) The FACS gate was deployed as shown.
C) The proportion of IgA⁺ cells among CD45⁺ cells in the large lamina propria (LLP) of each mouse using flow cytometry (Ctrl, n=5; L1w, n=7; L2w, n=4).
D, E) The proportions of neutrophils (D) and dendritic cells (E) among CD45+ cells in the salivary glands (SG) of each mouse using flow cytometry: neutrophils (Ctrl, n=6; L1w, n=6), dendritic cells (Ctrl, n=6; L1w, n=6). Neutrophils were gated with CD45+Gr-1+, while dendritic cells were gated with CD45+Gr-1−F4/80−CD11b+CD11c+. The experiment was repeated twice.
Data are expressed as mean ± SD. Statistical significance was determined using a two-tailed unpaired t-test. Statistical significance is indicated as follows: ns: p > 0.05.
Supplemental Figure 3. Effects on gut microbiome in a mouse model of experimental periodontitis.
Microbiome analysis was performed using feces from Ctrl and L1w group mice.
A) The microflora in the Ctrl and L1w groups at the family level.
B) The bacterial families significantly increased in the Ctrl or L1w group.
C, D) Alpha diversity (C) and beta diversity (D) in the gut microflora of the Ctrl and L1w groups. Alpha diversity was assessed using Chao1 and Shannon indices to evaluate the richness and evenness of the microbial communities.
Data are expressed as mean ± SD. Statistical significance was determined using a two-tailed unpaired t-test. Statistical significance is indicated as follows: *p < 0.05, ns: p > 0.05.

## Slide 3
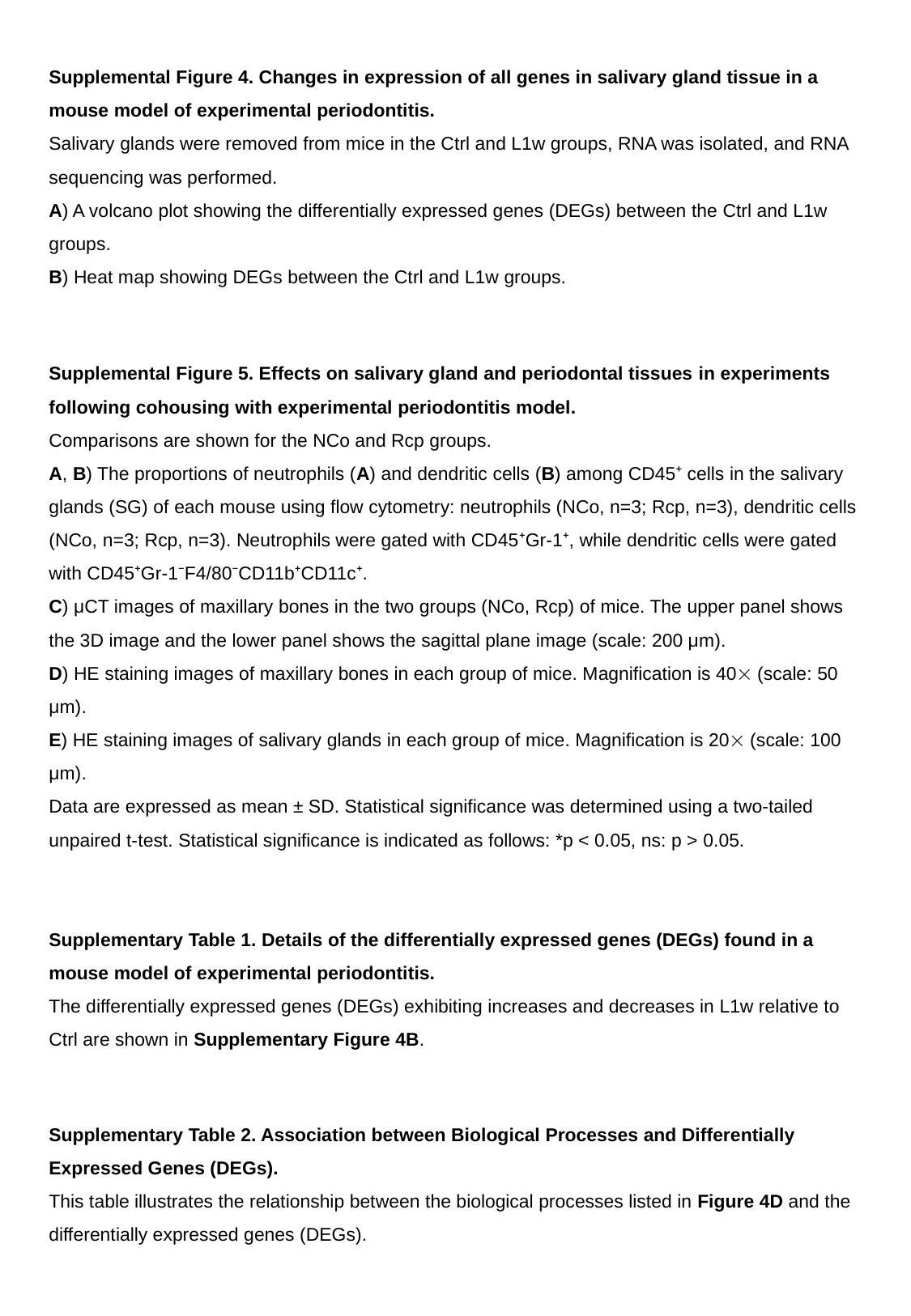

Supplemental Figure 4. Changes in expression of all genes in salivary gland tissue in a mouse model of experimental periodontitis.
Salivary glands were removed from mice in the Ctrl and L1w groups, RNA was isolated, and RNA sequencing was performed.
A) A volcano plot showing the differentially expressed genes (DEGs) between the Ctrl and L1w groups.
B) Heat map showing DEGs between the Ctrl and L1w groups.
Supplemental Figure 5. Effects on salivary gland and periodontal tissues in experiments following cohousing with experimental periodontitis model.
Comparisons are shown for the NCo and Rcp groups.
A, B) The proportions of neutrophils (A) and dendritic cells (B) among CD45⁺ cells in the salivary glands (SG) of each mouse using flow cytometry: neutrophils (NCo, n=3; Rcp, n=3), dendritic cells (NCo, n=3; Rcp, n=3). Neutrophils were gated with CD45⁺Gr-1⁺, while dendritic cells were gated with CD45⁺Gr-1⁻F4/80⁻CD11b⁺CD11c⁺.
C) μCT images of maxillary bones in the two groups (NCo, Rcp) of mice. The upper panel shows the 3D image and the lower panel shows the sagittal plane image (scale: 200 μm).
D) HE staining images of maxillary bones in each group of mice. Magnification is 40× (scale: 50 μm).
E) HE staining images of salivary glands in each group of mice. Magnification is 20× (scale: 100 μm).
Data are expressed as mean ± SD. Statistical significance was determined using a two-tailed unpaired t-test. Statistical significance is indicated as follows: *p < 0.05, ns: p > 0.05.
Supplementary Table 1. Details of the differentially expressed genes (DEGs) found in a mouse model of experimental periodontitis.
The differentially expressed genes (DEGs) exhibiting increases and decreases in L1w relative to Ctrl are shown in Supplementary Figure 4B.
Supplementary Table 2. Association between Biological Processes and Differentially Expressed Genes (DEGs).
This table illustrates the relationship between the biological processes listed in Figure 4D and the differentially expressed genes (DEGs).

## Slide 4
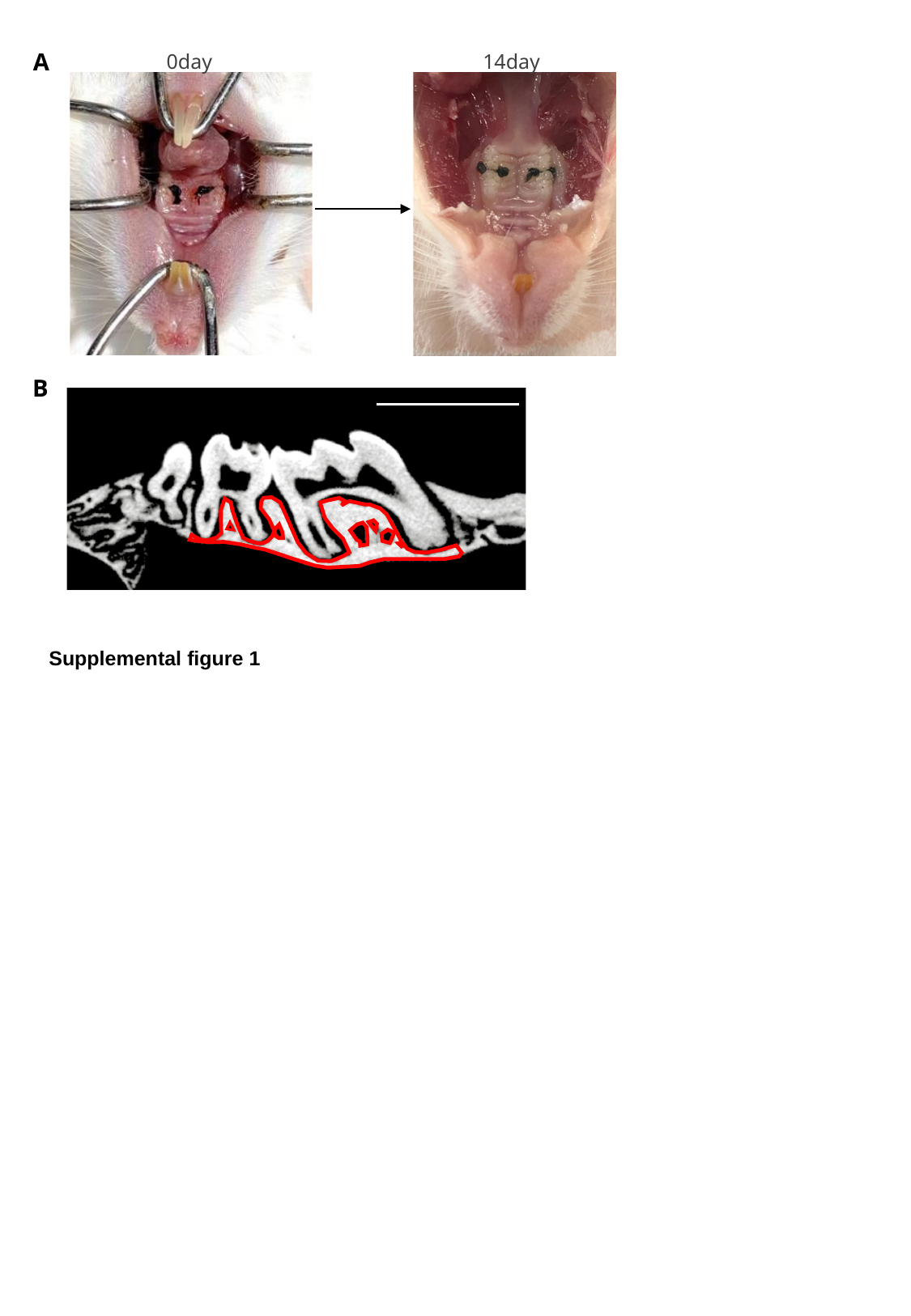

A
0day
14day
B
Supplemental figure 1

## Slide 5
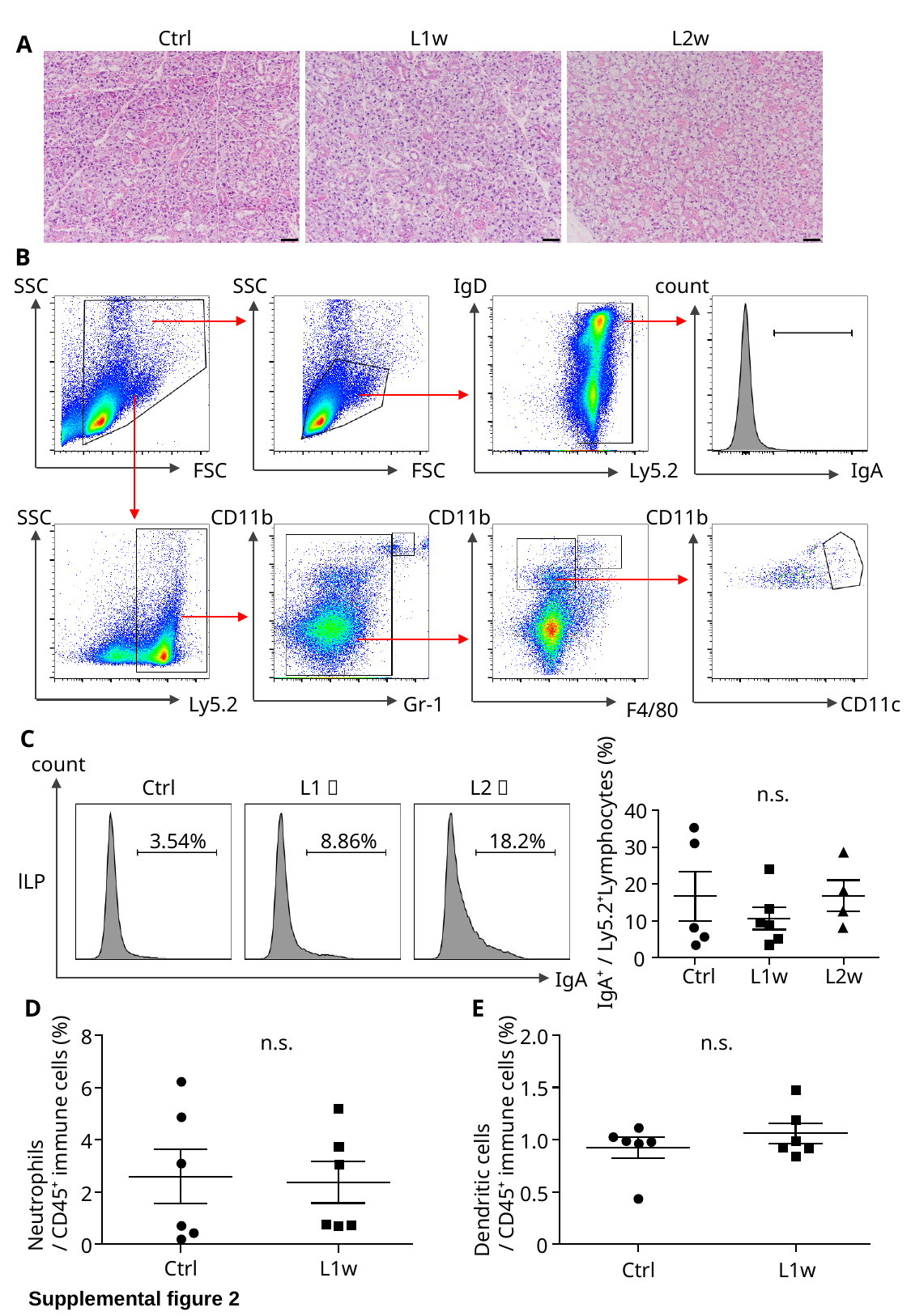

Ctrl
L1w
L2w
A
B
SSC
SSC
IgD
count
FSC
FSC
Ly5.2
IgA
SSC
CD11b
CD11b
CD11b
Ly5.2
Gr-1
CD11c
F4/80
C
n.s.
40
30
IgA⁺ / Ly5.2⁺Lymphocytes (%)
20
10
0
Ctrl
L1w
L2w
count
Ctrl
L1ｗ
L2ｗ
3.54%
8.86%
18.2%
lLP
IgA
D
E
2.0
1.5
Dendritic cells
 / CD45⁺ immune cells (%)
1.0
0.5
0
Ctrl
L1w
8
6
Neutrophils
/ CD45⁺ immune cells (%)
4
2
0
Ctrl
L1w
n.s.
n.s.
Supplemental figure 2

## Slide 6
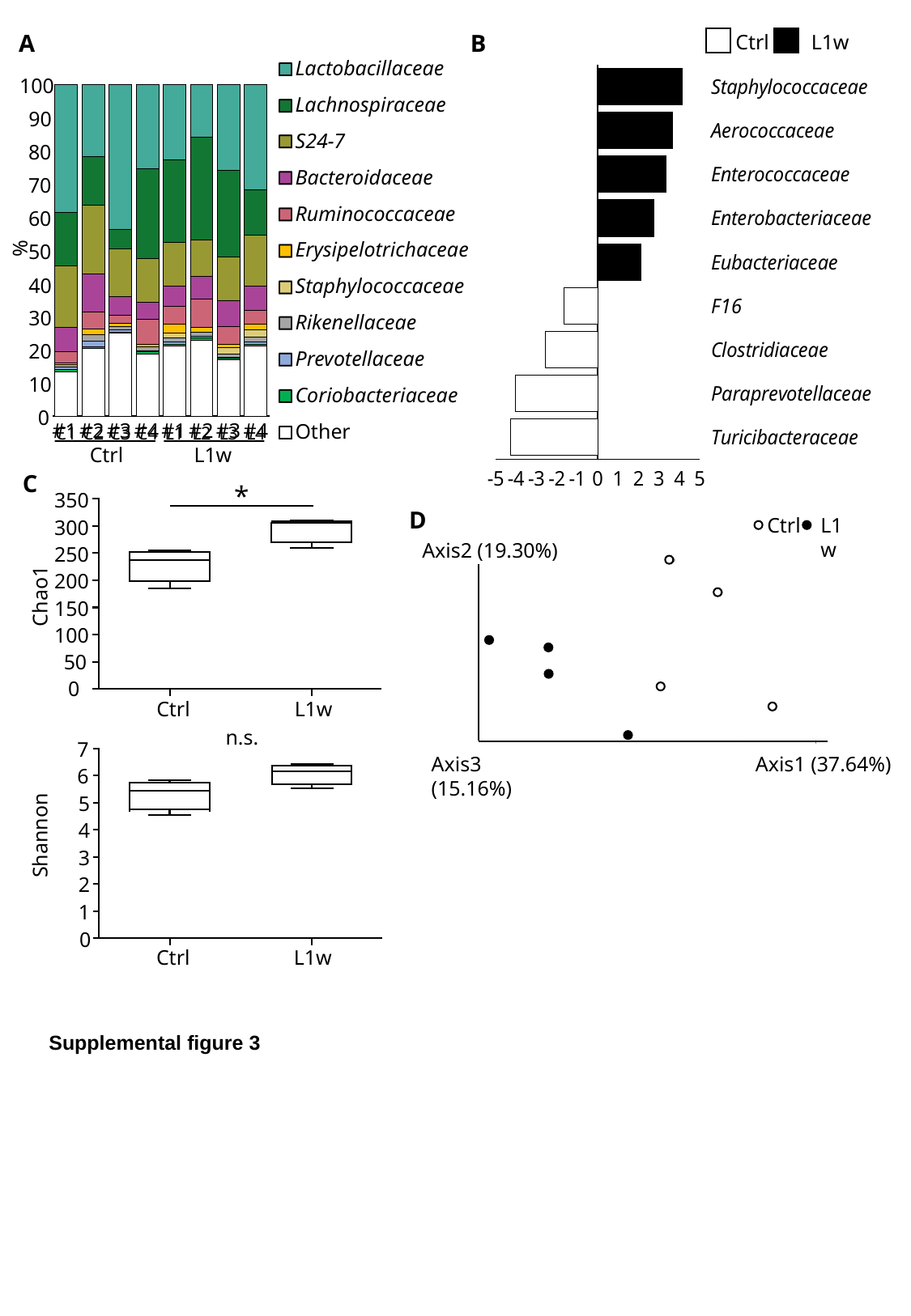

A
B
Ctrl
L1w
### Chart
| Category | |
|---|---|
| Turicibacteraceae | -4.28868393828 |
| Paraprevotellaceae | -4.02169706141 |
| Clostridiaceae | -2.57306084685 |
| F16 | -1.66954944091 |
| Eubacteriaceae | 2.13731689954 |
| Enterobacteriaceae | 2.75049216627 |
| Enterococcaceae | 3.35258532009 |
| Aerococcaceae | 3.64645892261 |
| Staphylococcaceae | 4.15347914353 |Lactobacillaceae
### Chart
| Category | other | Coriobacteriaceae | Prevotellaceae | Rikenellaceae | Staphylococcaceae | Erysipelotrichaceae | Ruminococcaceae | Bacteroidaceae | S24-7 | Lachnospiraceae | Lactobacillaceae |
|---|---|---|---|---|---|---|---|---|---|---|---|
| | 12106.0 | 719.0 | 617.0 | 589.0 | 6.0 | 531.0 | 3084.0 | 6688.0 | 16801.0 | 14456.0 | 34973.0 |
| | 16791.0 | 439.0 | 1252.0 | 1707.0 | 79.0 | 1392.0 | 4080.0 | 9555.0 | 17012.0 | 11884.0 | 17840.0 |
| | 17873.0 | 327.0 | 446.0 | 742.0 | 3.0 | 582.0 | 1900.0 | 3892.0 | 10395.0 | 4085.0 | 31334.0 |
| | 13874.0 | 529.0 | 208.0 | 856.0 | 0.0 | 628.0 | 5526.0 | 3844.0 | 9724.0 | 19962.0 | 18812.0 |
| | 18019.0 | 446.0 | 718.0 | 981.0 | 1246.0 | 2228.0 | 4720.0 | 5228.0 | 11276.0 | 21132.0 | 19368.0 |
| | 16526.0 | 483.0 | 365.0 | 824.0 | 80.0 | 1013.0 | 6145.0 | 4956.0 | 7909.0 | 22341.0 | 11454.0 |
| | 12639.0 | 300.0 | 201.0 | 722.0 | 1395.0 | 823.0 | 3929.0 | 5836.0 | 9650.0 | 19418.0 | 19086.0 |
| | 18574.0 | 331.0 | 640.0 | 1198.0 | 1956.0 | 1556.0 | 3669.0 | 6332.0 | 13492.0 | 11805.0 | 27776.0 |100
Lachnospiraceae
90
S24-7
80
Bacteroidaceae
70
Ruminococcaceae
60
Erysipelotrichaceae
50
40
Staphylococcaceae
30
Rikenellaceae
20
Prevotellaceae
10
Coriobacteriaceae
0
#1
#2
#3
#4
#1
#2
#3
#4
Other
Ctrl
L1w
C
*
350
300
250
200
Chao1
150
100
50
0
Ctrl
L1w
D
Ctrl
L1w
Axis2 (19.30%)
Axis3 (15.16%)
Axis1 (37.64%)
n.s.
7
6
5
4
Shannon
3
2
1
0
Ctrl
L1w
Supplemental figure 3

## Slide 7
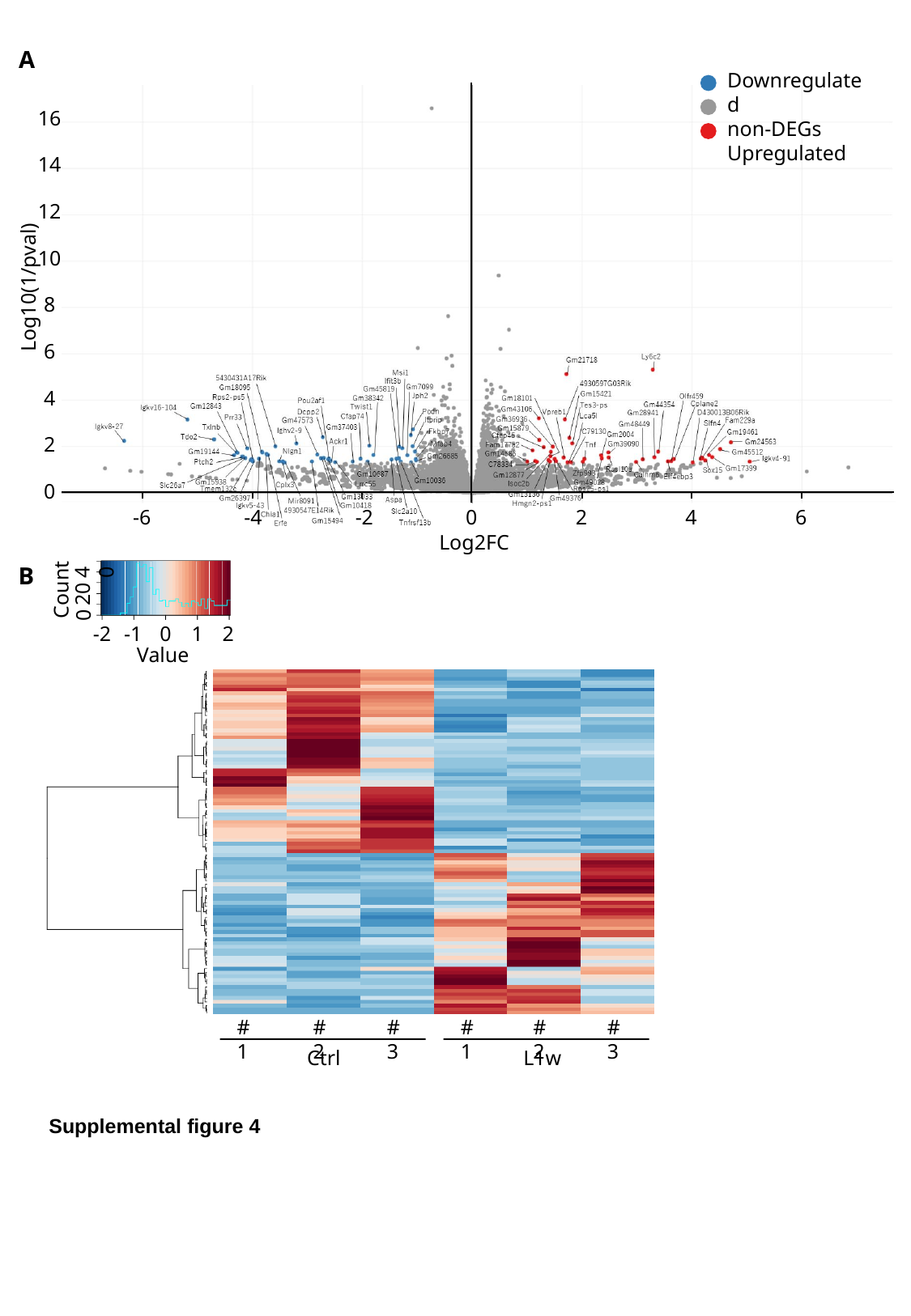

A
Downregulated
non-DEGs
Upregulated
16
14
12
10
Log10(1/pval)
8
6
4
2
0
-6
-4
-2
0
2
4
6
Log2FC
40
Count
20
0
-2
-1
0
1
2
Value
#1
#2
#3
#1
#2
#3
Ctrl
L1w
B
Supplemental figure 4

## Slide 8
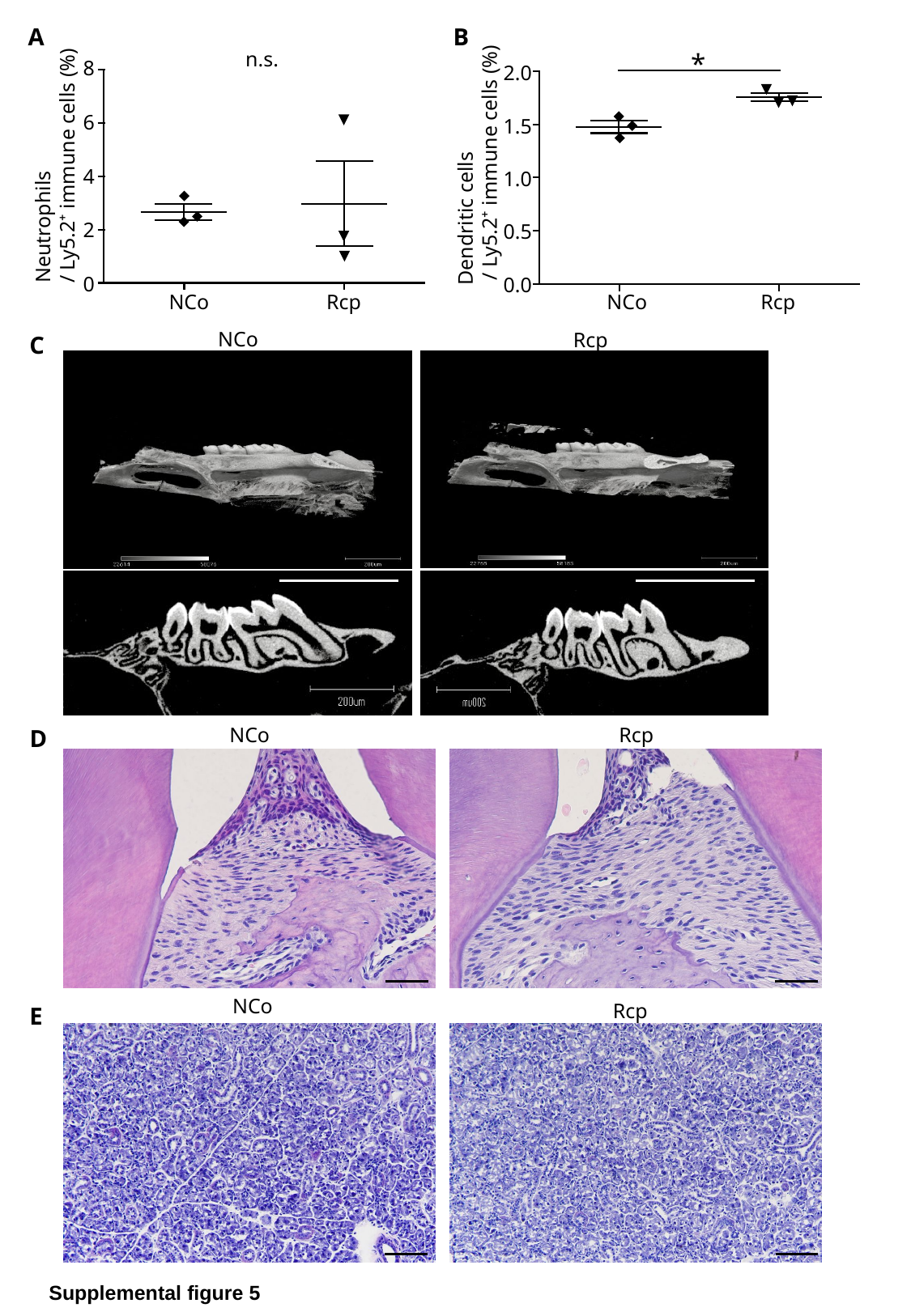

A
B
*
2.0
1.5
Dendritic cells
 / Ly5.2⁺ immune cells (%)
1.0
0.5
0.0
NCo
Rcp
n.s.
8
6
Neutrophils
/ Ly5.2⁺ immune cells (%)
4
2
0
NCo
Rcp
NCo
Rcp
C
Rcp
NCo
D
NCo
Rcp
E
Supplemental figure 5

## Slide 9
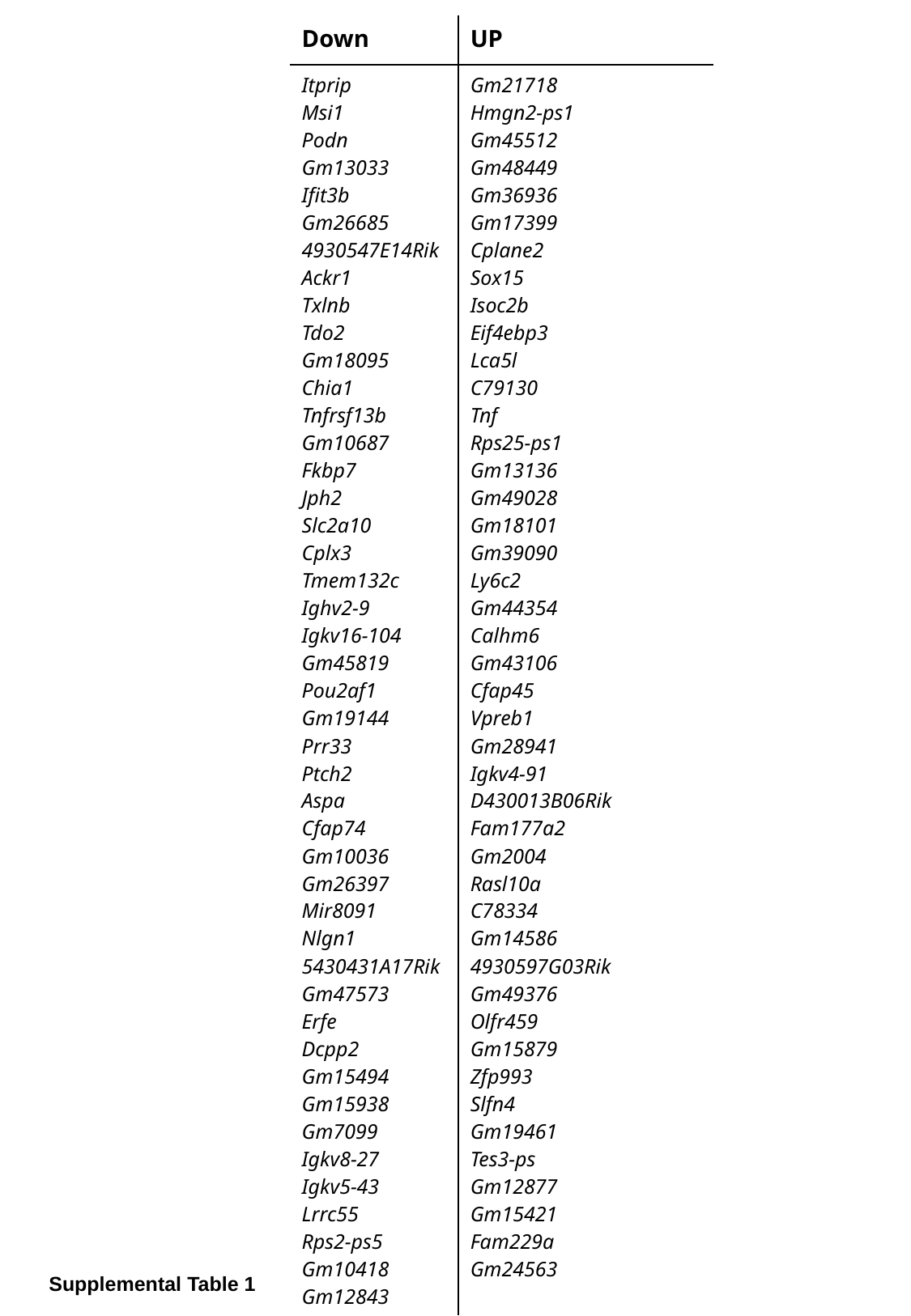

| Down | UP |
| --- | --- |
| Itprip Msi1 Podn Gm13033 Ifit3b Gm26685 4930547E14Rik Ackr1 Txlnb Tdo2 Gm18095 Chia1 Tnfrsf13b Gm10687 Fkbp7 Jph2 Slc2a10 Cplx3 Tmem132c Ighv2-9 Igkv16-104 Gm45819 Pou2af1 Gm19144 Prr33 Ptch2 Aspa Cfap74 Gm10036 Gm26397 Mir8091 Nlgn1 5430431A17Rik Gm47573 Erfe Dcpp2 Gm15494 Gm15938 Gm7099 Igkv8-27 Igkv5-43 Lrrc55 Rps2-ps5 Gm10418 Gm12843 Gm37403 Twist1 Mfap4 Gm38342 Slc26a7 | Gm21718 Hmgn2-ps1 Gm45512 Gm48449 Gm36936 Gm17399 Cplane2 Sox15 Isoc2b Eif4ebp3 Lca5l C79130 Tnf Rps25-ps1 Gm13136 Gm49028 Gm18101 Gm39090 Ly6c2 Gm44354 Calhm6 Gm43106 Cfap45 Vpreb1 Gm28941 Igkv4-91 D430013B06Rik Fam177a2 Gm2004 Rasl10a C78334 Gm14586 4930597G03Rik Gm49376 Olfr459 Gm15879 Zfp993 Slfn4 Gm19461 Tes3-ps Gm12877 Gm15421 Fam229a Gm24563 |
Supplemental Table 1

## Slide 10
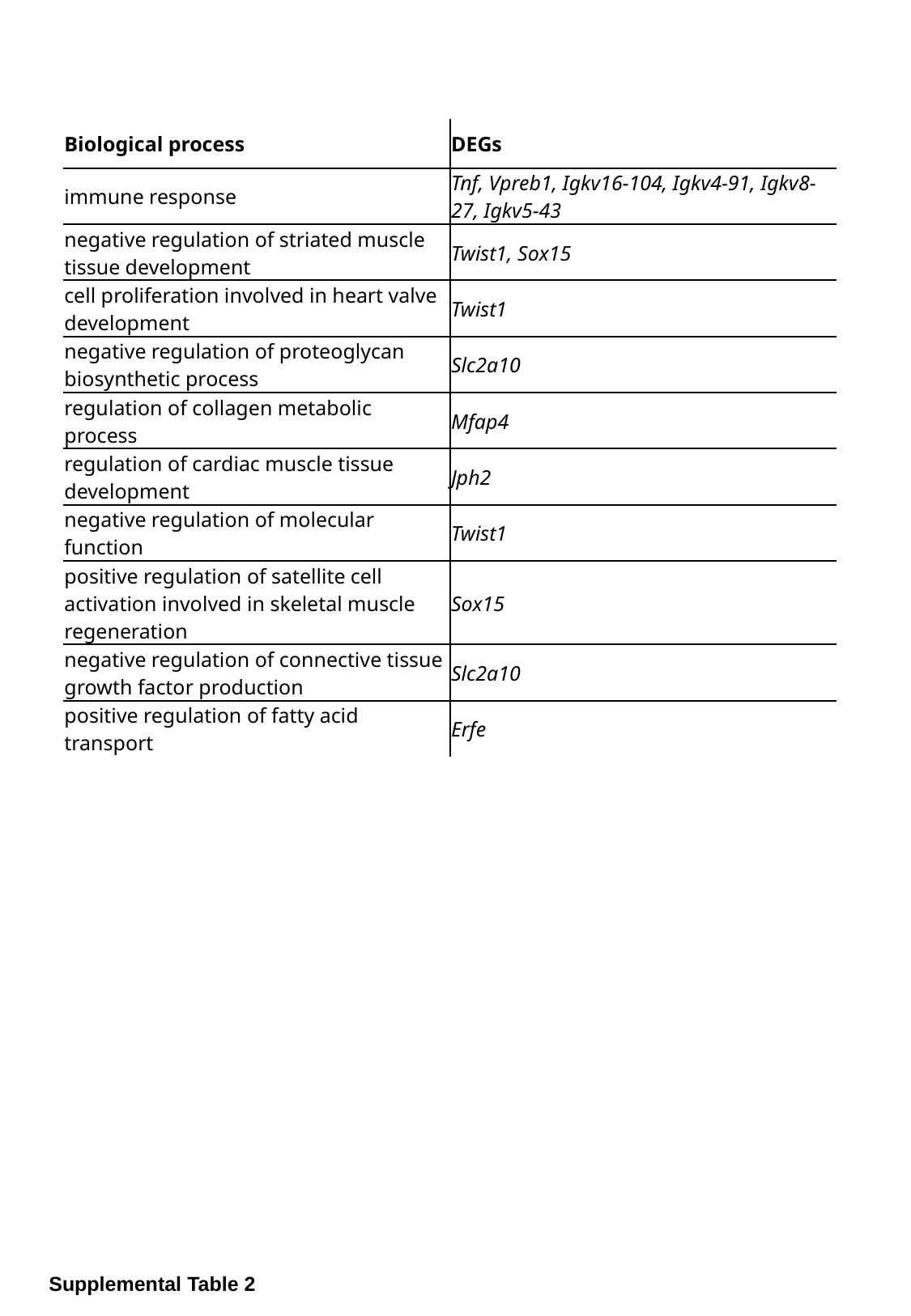

| Biological process | DEGs |
| --- | --- |
| immune response | Tnf, Vpreb1, Igkv16-104, Igkv4-91, Igkv8-27, Igkv5-43 |
| negative regulation of striated muscle tissue development | Twist1, Sox15 |
| cell proliferation involved in heart valve development | Twist1 |
| negative regulation of proteoglycan biosynthetic process | Slc2a10 |
| regulation of collagen metabolic process | Mfap4 |
| regulation of cardiac muscle tissue development | Jph2 |
| negative regulation of molecular function | Twist1 |
| positive regulation of satellite cell activation involved in skeletal muscle regeneration | Sox15 |
| negative regulation of connective tissue growth factor production | Slc2a10 |
| positive regulation of fatty acid transport | Erfe |
Supplemental Table 2
